# Impact of Pacemaker Lead Characteristics on Pacemaker Related Infection and Heart Perforation: A Nationwide Population-Based Cohort Study

**DOI:** 10.1371/journal.pone.0128320

**Published:** 2015-06-15

**Authors:** Yu-Sheng Lin, Tien-Hsing Chen, Sheng-Ping Hung, Dong Yi Chen, Chun-Tai Mao, Ming-Lung Tsai, Shih-Tai Chang, Chun-Chieh Wang, Ming-Shien Wen, Mien-Cheng Chen

**Affiliations:** 1 Division of Cardiology, Department of Internal Medicine, Chang Gung Memorial Hospital, Chiayi, Taiwan; 2 Graduate Institute of Clinical Medical Sciences, College of Medicine, Chang Gung University, Taoyuan, Taiwan; 3 Division of Cardiology, Chang-Gung Memorial Hospital, Linkou, Taiwan; 4 Chang Gung University College of Medicine, Taoyuan, Taiwan; 5 Section of Cardiology, Department of Medicine, Chang Gung Memorial Hospital, Keelung, Taiwan; 6 Division of Cardiology and Department of Internal Medicine, Kaohsiung Chang Gung Memorial Hospital, Chang Gung University College of Medicine, Kaohsiung, Taiwan; University of Minnesota, UNITED STATES

## Abstract

**Background:**

Several risk factors for pacemaker (PM) related complications have been reported. However, no study has investigated the impact of lead characteristics on pacemaker-related complications.

**Methods and Results:**

Patients who received a new pacemaker implant from January 1997 to December 2011 were selected from the Taiwan National Health Insurance Database. This population was grouped according to the pacemaker lead characteristics in terms of fixation and insulation. The impact of the characteristics of leads on early heart perforation was analyzed by multivariable logistic regression analysis, while the impact of the lead characteristics on early and late infection and late heart perforation over a three-year period were analyzed using Cox regression. This study included 36,104 patients with a mean age of 73.4±12.5 years. In terms of both early and late heart perforations, there were no significant differences between groups across the different types of fixation and insulations. In the multivariable Cox regression analysis, the pacemaker-related infection rate was significantly lower in the active fixation only group compared to either the both fixation (OR, 0.23; 95% CI, 0.07–0.80; *P* = 0.020) or the passive fixation group (OR, 0.26; 95% CI, 0.08–0.83; *P* = 0.023).

**Conclusions:**

There was no difference in heart perforation between active and passive fixation leads. Active fixation leads were associated with reduced risk of pacemaker-related infection.

## Introduction

The pacemaker (PM) is standard therapy for bradyarrhythmias [1,2] and the population with implanted devices continues to grow [3], PM-related complications such as infection, and cardiac perforation will increase as well [4,5]. These complications not only result in prolonged hospitalization and increased costs, but also accrue worse outcomes and mortality [6]. For these reasons, many studies have attempted to investigate factors causing PM-related complications by evaluating baseline characteristics, implant procedures, and medications. Among the evaluated risks of the PM-related infection, several risk factors have been reported, such as diabetes mellitus, end-stage renal disease, corticosteroid use, and so on [7–11]. Although heart perforation is a relatively rare PM-related complication with a reported incidence ranging from 0.09% to 1.2% in the literature [12], some studies have pointed out certain risk factors for heart perforation such as temporary pacemakers, steroid use within 7 days prior to implantation, and helical screws [13].

Although a number of studies have reported many risk factors contributing to PM-related complications [13,14], most of which being mechanical complications, the relationship between the characteristics of pacemaker leads (in terms of fixation types or insulation materials) and PM-related complications remains unclear. Only a handful of case report studies [15] have mentioned this relationship. Accordingly, this study investigated the relationship between the characteristics of PM leads, fixation type and insulation, and PM-related complications in a large number of patients from a nationwide database

## Materials and Methods

### Data Source

This retrospective national population-based cohort study was retrieved from the National Health Insurance Research Database (NHIRD) released by the Taiwan National Health Research Institute (NHRI) *(* *http*:*//nhird*.*nhri*.*org*.*tw/en/index*.*htm* *)*. The data of NHIRD contain registration files and original claim data for reimbursement, which were derived from the National Health Insurance Administration, Ministry of Health and Welfare and maintained by the NHRI. The NHIRD contains health care information of the 99.9% of the Taiwanese population (about 23.20 million in 2012) enrolled in the NHI program [16]. Previous studies have described the NHIRD in detail and validated the accuracy of its diagnostic data [17]. The insurance has since 1997 reimbursed all the new implantation, replacement, revision and removal expenses of cardiac implantable electronic devices (CIEDs), with the appropriate indications according to the clinical practice guidelines of the CIED. In addition, the expenses of the insurance include all the generators, CIED leads and physician fees. The original data of the NHIRD are unstructured data, which all the contents are string variables and are not able to be analyzed directly. According to the coded book provided by the NHRI (http://nhird.nhri.org.tw/date_02.htm), the original unstructured data were transformed into structured data, which were composed of numeric variables by using SAS Version 9.3 (SAS Institute, Cary, NC). Analyzing statistical analysis becomes feasible when the data are numeric rather than string type.

### Study design

**Fig 1:**
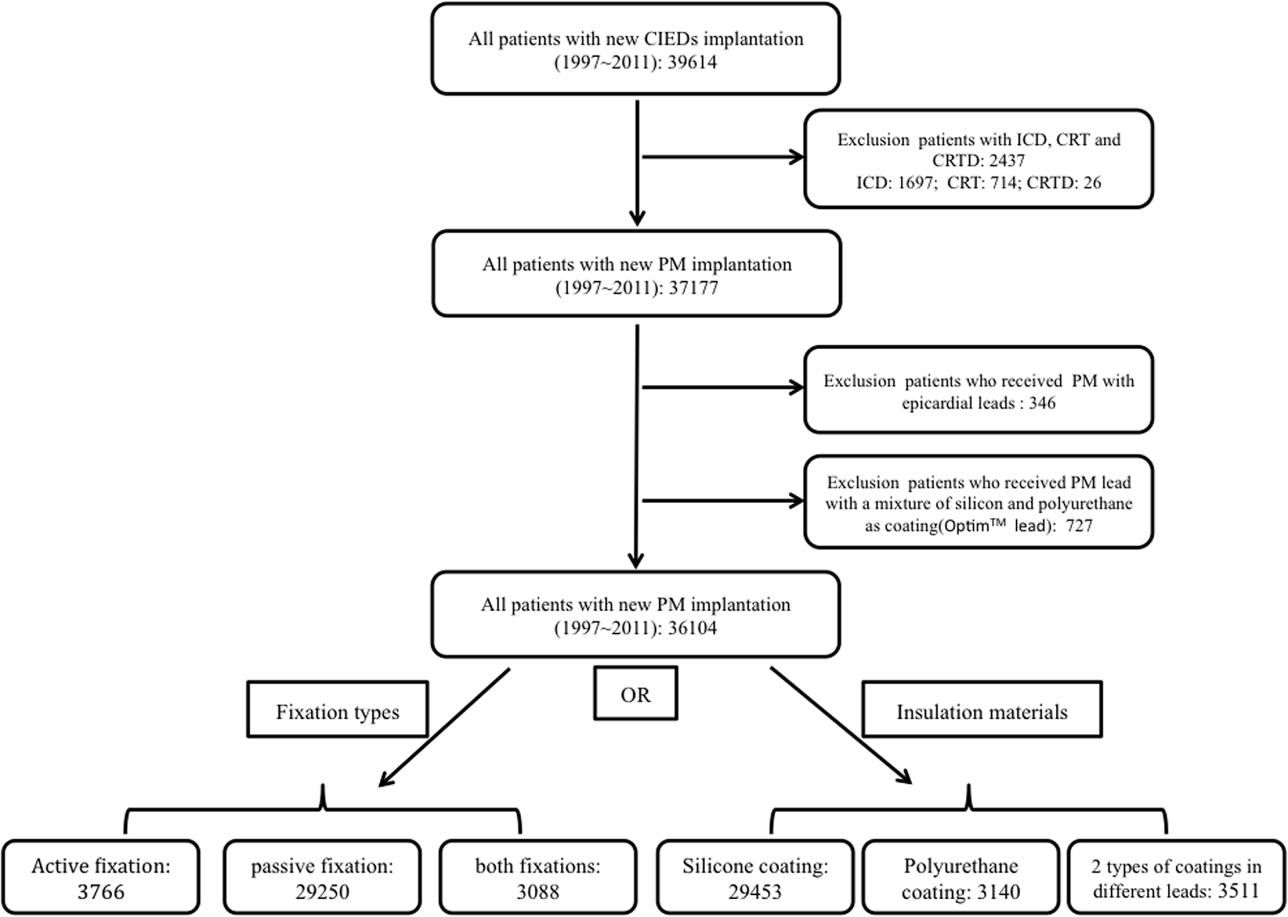
Study design and flow chart of patient selection. CIED = cardiac implantable electronic devices; ICD = implantable cardiac defibrillator; CRT = cardiac resynchronization therapy; CRTD = cardiac resynchronization therapy defibrillator; PM = pacemaker.

In the initial selection of the population set, the patients who received CIED implantation between January 1, 1997 and December 31, 2011 were included (Fig 1). We excluded patients who received implantable cardiac defibrillators, cardiac resynchronization therapy and cardiac resynchronization therapy defibrillator implantation. Furthermore, patients who received PM with epicardial leads were also excluded in order to reduce the procedure related bias. Moreover, we excluded patients who received leads with a mixture of silicone and polyurethane as insulation (Optim). Finally, we analyzed the impact of the fixation types or insulation materials of the leads on PM-related complications (Fig 1).

There were two outcomes specifically evaluated in this study. One was heart perforation that included early and late heart perforation; the other was PM-related infection, which included early and late infection and infection that either needed lead extraction or without lead extraction. This cohort study was followed up for three years after the index date. In the cohort dataset from NHIRD, the patients' original identification numbers have been encrypted to protect their privacy and the encrypted data were for research purposes only. We used NHIRD data set for this study and obtained ethical approval from the Institutional Review Board of Chang Gung Memorial Hospital (101-2055B).

### Definitions

PM-related heart perforation was defined as a heart perforation treated with heart repair during admission for any implantation procedure, replacement, revision, or removal. Early heart perforation was defined as the events occurring within one month [12] after implantation, while late heart perforation was defined as events developing more than one month after implantation. Pacemaker infection was defined as an infection (S1 Table) that occurred during admission for implantation, replacement, revision, or removal. Early infection was defined as the PM-related infection developing within one year after the new implantation, while late infection was defined as PM-related infection developing more than one year after the new implantation [9].

The analyzed characteristics of leads were fixation types and insulation materials. In the analysis of fixation type, the population was divided into the three groups of active fixation (screw lead), passive (tined lead) and both-types, which meant that one lead was active fixation and the other was passive fixation in the dual chamber pacemaker. In the analysis of insulation, the population was divided into the three groups of silicone coating, polyurethane coating and both-coating which meant that one lead had a silicone coating and the other a polyurethane coating in the dual chamber pacemaker.

### Statistical Analysis

The clinical characteristics of the study participants are presented as number and percentage for categorical variables or as mean and standard deviation for continuous variables. The association between study groups (different fixation types and insulation types) and early heart perforation was examined using multivariable logistic regression analysis. The three-year event-free survival rates of late heart perforation and PM-related infection among the study groups were compared using a multivariable Cox proportional hazard analysis. The results are presented as an adjusted odds ratio (OR) for logistic regression or adjusted hazard ratio (HR) for Cox regression with its corresponding 95% confidence intervals (CI). To rule out the confounding effects, both logistic and Cox regression analyses were performed with adjustment of patient’s characteristics, such as gender, age group, hospital level, device type, diabetes mellitus, liver cirrhosis, obstructive lung disease, chronic kidney disease, heart failure, hypertension, coronary artery disease, malignant neoplasm, antibiotics use, steroid use, and combination of warfarin and antiplatelet use. Data analyses were conducted using SPSS software version 15.0 (SPSS Inc., Chicago, IL, USA).

## Results

### Patient Characteristics

There were 36,104 PM patients with the mean age of 73.4 years (SD = 12.5 years) (range: 8 years to 105 years) were enrolled in this study and the majority of the population were older than 70 years. When the population was divided into three groups according to the fixation types, pacemakers with only active fixation leads were implanted in 3,766 patients, with only passive fixation leads in 29,250 patients, and with both fixation leads in 3,088 patients (Table 1). When the population was divided into three groups by different insulation of leads, the pacemakers with silicone-coating leads were implanted in 29,453 patients, with polyurethane-coating leads in 3,140 patients, and with one silicone-coating and the other polyurethane-coating leads in 3,511 patients (Table 1). There were similar distributions in age-scale, gender, comorbidities in each group and nearly all patients were prescribed intravenous antibiotics during the procedures. Furthermore, in the passive fixation group, most of the patients received a single chamber PM, while in the active fixation group, most received a dual chamber PM.

**Table 1:** Patient Characteristics at Implant Stratified by Lead Fixation Method and Insulation (*n* = 36,104).

	Fixation method				Insulation			
Variable	Active fixation	Passive fixation	Both fixation	*P* Value	Silicone	Polyurethane	Both insulation	*P* Value
Number of patient	3,766	29,250	3,088	—	29,453	3,140	3,511	—
Gender––no. (%)				<0.001^a^ ^b^				0.001^d^ ^f^
Male	2,058 (54.6)	14,871 (50.8)	1,518 (49.2)		15,142 (51.4)	1,502 (47.8)	1,803 (51.4)	
Female	1,708 (45.4)	14,379 (49.2)	1,570 (50.8)		14,311 (48.6)	1,638 (52.2)	1,708 (48.6)	
Age––yr±SD	74.6±11.8	73.1±12.7	74.7±10.5	<0.001	73.4±12.4	73.5±12.6	73.5±12.6	0.849
Age group––no. (%)				<0.001^a^ ^b^ ^c^				0.003^e^
< 20 yrs	14 (0.4)	252 (0.9)	4 (0.1)		229 (0.8)	19 (0.6)	22 (0.6)	
20~49 yrs	130 (3.5)	1,090 (3.7)	77 (2.5)		1,019 (3.5)	130 (4.1)	148 (4.2)	
50~59 yrs	258 (6.9)	2,013 (6.9)	203 (6.6)		2,005 (6.8)	202 (6.4)	267 (7.6)	
60~69 yrs	597 (15.9)	5,504 (18.8)	533 (17.3)		5,483 (18.6)	553 (17.6)	598 (17.0)	
70~79 yrs	1,431 (38.0)	11,676 (39.9)	1,262 (40.9)		11,769 (40.0)	1,264 (40.3)	1,336 (38.1)	
>80 yrs	1,336 (35.5)	8,715 (29.8)	1,009 (32.7)		8,948 (30.4)	972 (31.0)	1,140 (32.5)	
Device type––no. (%)				<0.001^a^ ^b^ ^c^				<0.001^d^ ^e^ ^f^
Single chamber PPM	547 (14.5)	21,567 (73.7)	0 (0.0)		19,566 (66.4)	2,548 (81.1)	0 (0.0)	
Dual chamber PPM	3,219 (85.5)	7,683 (26.3)	3,088 (100.0)		9,887 (33.6)	592 (18.9)	3,511 (100.0)	
Indication––no. (%)								
AV block	1,462 (38.8)	10,517 (36.0)	986 (31.9)	<0.001^a^ ^b^ ^c^	10,405 (35.3)	1,158 (36.9)	1,402 (39.9)	<0.001^e^ ^f^
Congenital AV block	1 (0.0)	60 (0.2)	0 (0.0)	0.002^a^ ^c^	57 (0.2)	3 (0.1)	1 (0.0)	0.046^e^
AF	1,016 (27.0)	5,842 (20.0)	667 (21.6)	<0.001^a^ ^b^ ^c^	6,038 (20.5)	610 (19.4)	877 (25.0)	<0.001^e^ ^f^
Sick sinus syndrome	1,813 (48.1)	14,742 (50.4)	1,726 (55.9)	<0.001^a^ ^b^ ^c^	14,956 (50.8)	1,620 (51.6)	1,705 (48.6)	0.024^e^ ^f^
Comorbidity––no. (%)								
Diabetes	1,235 (32.8)	8,139 (27.8)	947 (30.7)	<0.001^a^ ^c^	8,236 (28.0)	970 (30.9)	1,115 (31.8)	<0.001^d^ ^e^
Liver cirrhosis	103 (2.7)	808 (2.8)	89 (2.9)	0.920	807 (2.7)	100 (3.2)	93 (2.6)	0.317
Obstructive lung disease	401 (10.6)	2,491 (8.5)	318 (10.3)	<0.001^a^ ^c^	2,561 (8.7)	309 (9.8)	340 (9.7)	0.022^d^
CKD	486 (12.9)	3,216 (11.0)	362 (11.7)	0.002^a^	3,208 (10.9)	414 (13.2)	442 (12.6)	<0.001^d^ ^e^
Heart failure	547 (14.5)	3,773 (12.9)	376 (12.2)	0.007^a^ ^b^	3,812 (12.9)	378 (12.0)	506 (14.4)	0.012^e^ ^f^
Hypertension	2,701 (71.7)	18,500 (63.2)	2,169 (70.2)	<0.001^a^ ^c^	18,846 (64.0)	2,041 (65.0)	2,483 (70.7)	<0.001^e^ ^f^
CAD	1,412 (37.5)	10,365 (35.4)	1,288 (41.7)	<0.001^a^ ^b^ ^c^	10,588 (35.9)	1,234 (39.3)	1,243 (35.4)	0.001^d^ ^f^
Malignant neoplasm	530 (14.1)	4,217 (14.4)	459 (14.9)	0.650	4,239 (14.4)	501 (16.0)	466 (13.3)	0.008^d^ ^f^
Medication––no. (%)								
Antibiotics	3,653 (97.0)	28,216 (96.5)	2,952 (95.6)	0.007^b^ ^c^	28,415 (96.5)	2,993 (95.3)	3,413 (97.2)	<0.001^d^ ^e^ ^f^
Warfarin	362 (9.6)	2,205 (7.5)	202 (6.5)	<0.001^a^ ^b^ ^c^	2,192 (7.4)	243 (7.7)	334 (9.5)	<0.001^e^ ^f^
Steroid	447 (11.9)	3,120 (10.7)	358 (11.6)	0.033^a^	3,065 (10.4)	445 (14.2)	415 (11.8)	<0.001^d^ ^e^ ^f^
Anti-platelet	1,638 (43.5)	12,161 (41.6)	1,380 (44.7)	0.001^a^ ^c^	12,075 (41.0)	1,565 (49.8)	1,539 (43.8)	<0.001^d^ ^e^ ^f^
Hospital level––no. (%)				<0.001^a^ ^b^ ^c^				<0.001^d^ ^f^
Medical center	2,347 (62.3)	16,821 (57.5)	1,382 (44.8)		16,999 (57.7)	1,524 (48.5)	2,027 (57.7)	
Metropolitan	1,355 (36.0)	11,667 (39.9)	1,591 (51.5)		11,652 (39.6)	1,556 (49.6)	1,405 (40.0)	
Local community	64 (1.7)	762 (2.6)	115 (3.7)		802 (2.7)	60 (1.9)	79 (2.3)	

Data are presented as mean ± SD or number (percentage).

AF = atrial fibrillation; AV block = atrioventricular block; CAD = coronary artery disease; CKD = chronic kidney disease; PPM = permanent pacemaker; SD = standard deviation

^a^ *P* < 0.05 for active fixation *vs*. passive fixation

^b^ *P* < 0.05 for active fixation *vs*. both fixation

^c^ *P* < 0.05 for passive fixation *vs*. both fixation

^d^ *P*< 0.05 for silicon *vs*. polyurethane

^e^ *P* < 0.05 for silicon *vs*. both

^f^ *P* < 0.05 for polyurethane *vs*. both.

### Pacemaker-Related Heart Perforation

**Fig 2:**
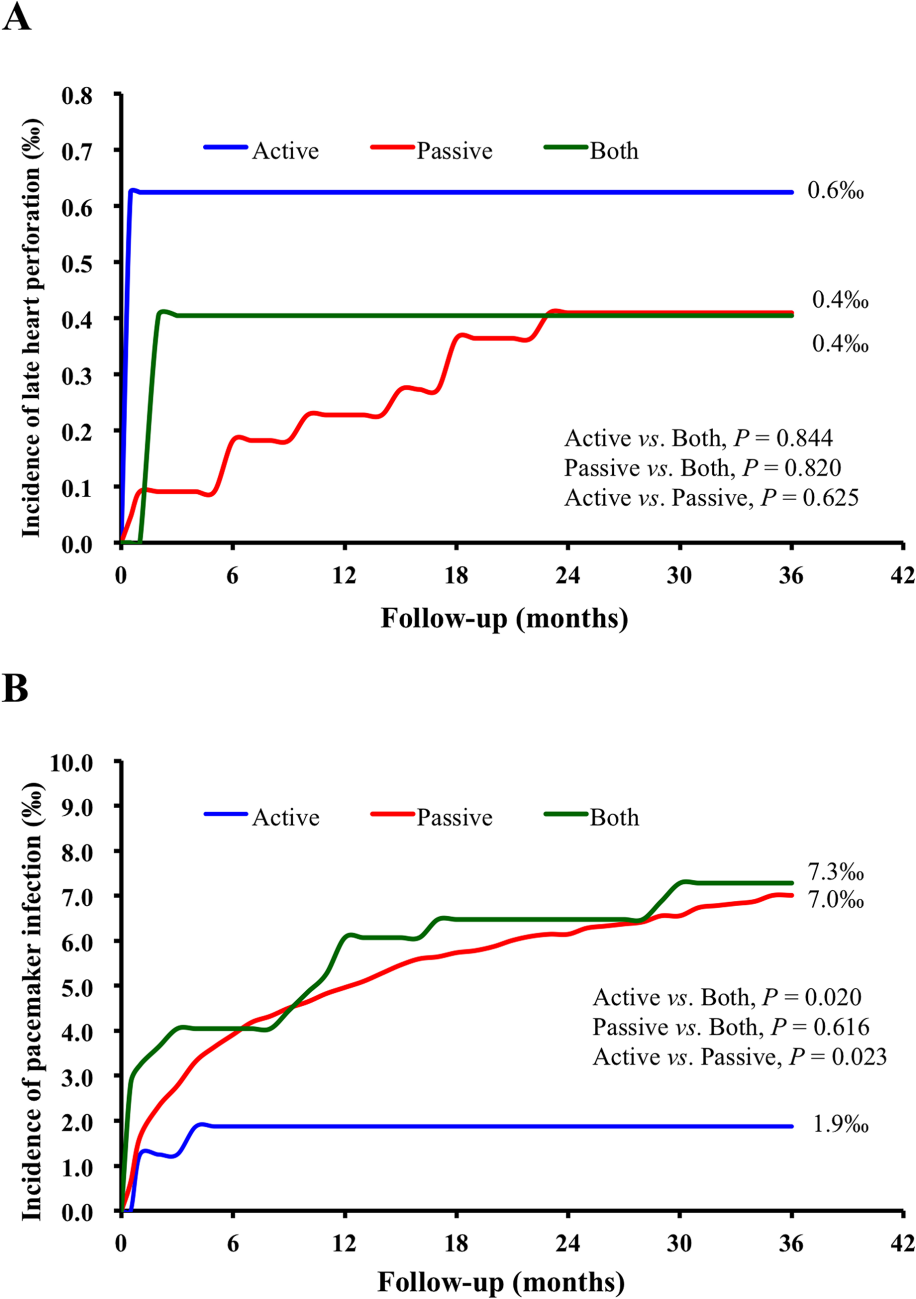
Kaplan–Meier estimates of the cumulative incidence of complications with different fixation types during three years of follow-up. (**A**) The cumulative incidence of late heart perforation; (**B**) The cumulative incidence of early and late complications of pacemaker infection. The blue line indicates active fixation group, red line the passive fixation group, and the green line the both-fixation group.

**Fig 3:**
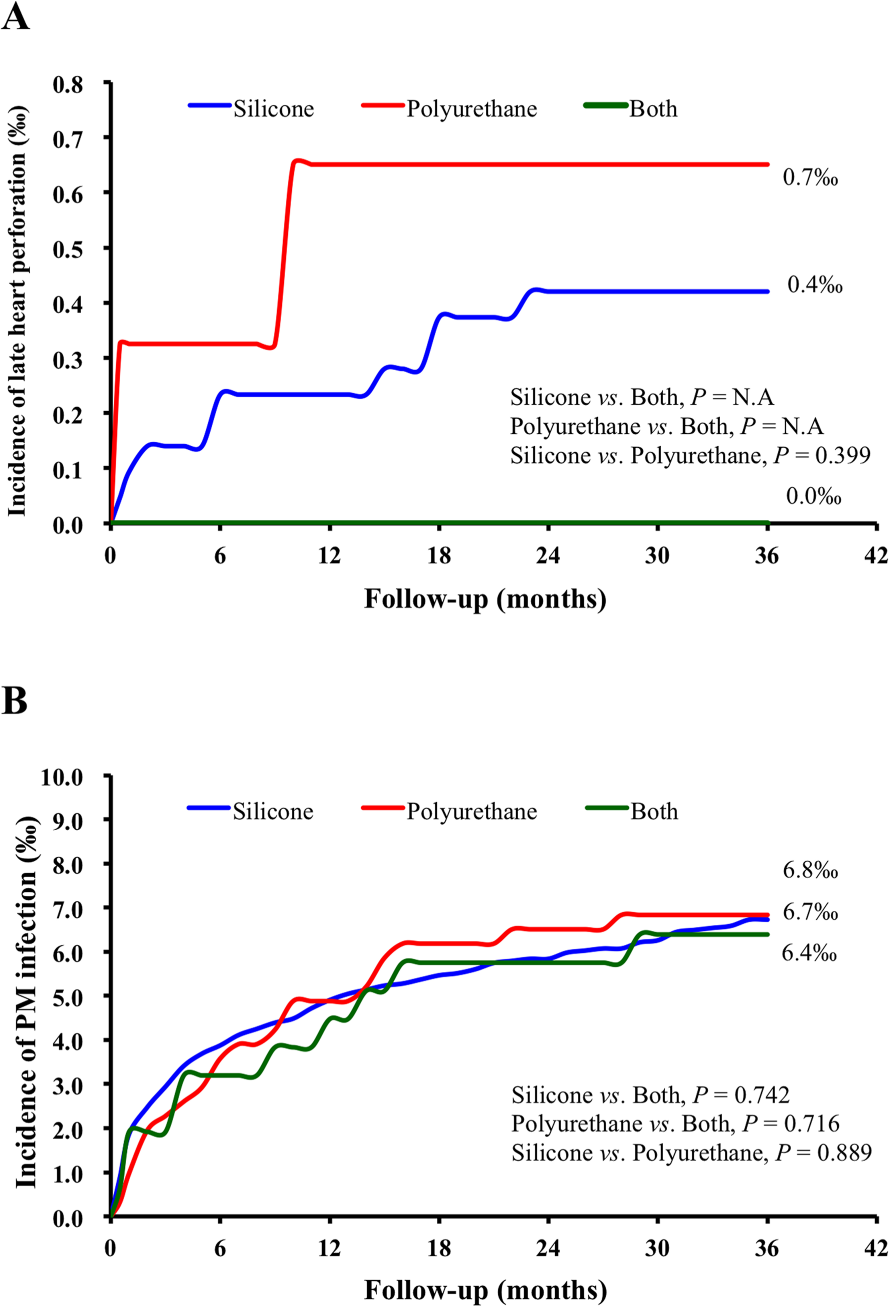
Kaplan–Meier estimates of the cumulative incidence of complications with different insulation materials during three years follow-up. (**A**) The cumulative incidence of late heart perforation; (**B**) The cumulative incidence of early and late complications of pacemaker infection. The blue line indicates the silicone group, the red line the polyurethane group, and the green line the both-coatings group.

Regarding early heart perforation, during the 14-year study period, there were 22 heart perforation (0.06%) events. In the multivariable logistic regression analysis, the incidence of early heart perforation was not significantly different between groups with different fixation methods, and there were also no significant differences between groups with different insulations (Table 2). Regarding the late heart perforation, the incidence of complications did not differ year by year according to the new implant year (S2 and S3 Tables). In the 3-year follow-up period, the incidence of late heart perforation was 0.04% (11 out of 26,047). The cumulative incidence of late heart perforation did not differ for the different types of fixation (Fig 2 and Table 3) and insulations (Fig 3 and Table 4).

**Table 2:** Lead Fixation Method and Lead Insulation *vs*. Early Heart Perforation Under Logistic Regression Analysis.

	Number of Event––no. (%)			Adjusted Odds Ratio and 95% CI					
	Active	Passive	Both	Active *vs*. Both		Passive *vs*. Both		Active *vs*. Passive	
**Outcome**	**(*n* = 3,766)**	**(*n* = 29,250)**	**(*n* = 3,088)**	**OR (95% CI)**	***P***	**OR (95% CI)**	***P***	**OR (95% CI)**	***P***
**Early heart perforation**	3 (0.08)	17 (0.06)	2 (0.06)	1.16 (0.19–7.23)	0.870	0.66 (0.11–3.93)	0.647	1.77 (0.41–7.54)	0.441
	**Silicone**	**Polyurethane**	**Both Insulation**	**Silicone *vs*. Both Insulation**		**Polyurethane *vs*. Both Insulation**		**Silicone *vs*. Polyurethane**	
**Outcome**	**(*n* = 29,453)**	**(*n* = 3,140)**	**(*n* = 3,511)**	**OR (95% CI)**	***P***	**OR (95% CI)**	***P***	**OR (95% CI)**	***P***
**Early heart perforation**	16 (0.05)	2 (0.06)	4 (0.11)	0.54 (0.18–1.64)	0.275	0.47 (0.08–2.60)	0.384	1.15 (0.26–5.10)	0.850

The odds ratios (OR) were adjusted for all covariates listed in Table 1.

**Table 3:** Lead Fixation Method *vs*. Late Heart Perforation and Infection Under Cox Logistic Regression Analysis.

	Number of Event––no. (%)			Adjusted Hazard Ratio and 95% CI					
	Active	Passive	Both	Active *vs*. Both		Passive *vs*. Both		Active *vs*. Passive	
**Outcome**	**(*n* = 1,602)**	**(*n* = 21,973)**	**(*n* = 2,472)**	**HR (95% CI)**	***P***	**HR (95% CI)**	***P***	**HR (95% CI)**	***P***
Late heart perforation	1 (0.06)	9 (0.04)	1 (0.04)	1.33 (0.08–22.02)	0.844	0.79 (0.10–6.26)	0.820	1.69 (0.21–13.77)	0.625
Infection	3 (0.19)	154 (0.70)	18 (0.73)	0.23 (0.07–0.80)	0.020	0.88 (0.54–1.44)	0.616	0.26 (0.08–0.83)	0.023
Early *vs*. late									
Early infection	3 (0.19)	106 (0.48)	13 (0.53)	0.31 (0.09–1.08)	0.066	0.85 (0.47–1.51)	0.571	0.36 (0.11–1.14)	0.083
Late infection	0 (0.00)	48 (0.22)	5 (0.20)	NA	NA	0.99 (0.39–2.50)	0.977	NA	NA
Need of lead-extraction *vs*. without lead extraction									
Need of lead extraction	2 (0.12)	90 (0.41)	13 (0.53)	0.24 (0.05–1.06)	0.060	0.77 (0.43–1.39)	0.394	0.31 (0.08–1.25)	0.099
Without lead extraction	1 (0.06)	64 (0.29)	5 (0.20)	0.24 (0.03–2.04)	0.189	1.17 (0.47–2.93)	0.734	0.20 (0.03–1.46)	0.112

The hazard ratios (HR) were adjusted for all covariates listed in Table 1.

**Table 4:** Lead Insulation *vs*. Late Heart Perforation and Infection Under Cox Logistic Regression Analysis.

	Number of Event––no. (%)			Adjusted Hazard Ratio and 95% CI					
	Silicone	Polyurethane	Combined	Silicone *vs*. Combined		Polyurethane *vs*. Combined		Silicone *vs*. Polyurethane	
**Outcome**	**(*n* = 21,409)**	**(*n* = 3,073)**	**(*n* = 1,565)**	**HR (95% CI)**	***P***	**HR (95% CI)**	***P***	**HR (95% CI)**	***P***
Late heart perforation	9 (0.04)	2 (0.07)	0 (0.00)	NA	NA	NA	NA	0.51 (0.11–2.44)	0.399
Infection	144 (0.67)	21 (0.68)	10 (0.64)	1.11 (0.58–2.13)	0.742	1.15 (0.54–2.47)	0.716	0.97 (0.61–1.54)	0.889
Early *vs*. late									
Early infection	101 (0.47)	15 (0.49)	6 (0.38)	1.38 (0.60–3.16)	0.447	1.43 (0.55–3.72)	0.467	0.97 (0.56–1.67)	0.906
Late infection	43 (0.20)	6 (0.20)	4 (0.26)	0.73 (0.26–2.06)	0.553	0.76 (0.21–2.77)	0.683	0.95 (0.40–2.26)	0.915
Need of lead-extraction *vs*. without lead extraction									
Need of lead-extraction	82 (0.38)	17 (0.55)	6 (0.38)	1.05 (0.45–2.41)	0.915	1.51 (0.59–3.88)	0.390	0.69 (0.41–1.17)	0.172
Without lead-extraction	62 (0.29)	4 (0.13)	4 (0.26)	1.25 (0.45–3.47)	0.670	0.61 (0.15–2.47)	0.487	2.06 (0.74–5.68)	0.165

The hazard ratios (OR) were adjusted for all covariates listed in Table 1.

### Pacemaker-Related Infection

During the 3-year follow-up period, the incidence of PM-related infection was 0.67% (175 out of 26,047). In the multivariable Cox-regression analysis, the PM-related cumulative infection rate was significantly lower in the active fixation group compared to either the both fixation group (OR, 0.23; 95% CI, 0.07–0.80; *P* = 0.020) or the passive fixation group (OR, 0.26; 95% CI, 0.08–0.83; *P* = 0.023) (Fig 2 and Table 3). In the subgroup analysis, the active fixation group had a lower early PM-related infection rate and a lower rate of PM-related infection that required lead extractions compared to either the both fixation group or the passive fixation group (Table 3). Moreover, there was no late infection (0%) in the active fixation group compared to 0.20% in the both fixation group and 0.22% in the passive fixation group (Table 3).

Regarding the insulations, the types of lead insulation had no impact on the cumulative incidence of PM-related infection (Fig 3 and Table 4). In the subgroup analysis, the insulation types of leads also did not differ in terms of the incidence of early and late infection and infection that required lead extraction or infection without lead extraction (Table 4).

## Discussion

This is the first study to investigate the impact of characteristics of leads on the major PM-related complications. In this nationwide cohort study, there was no difference in heart perforations between active and passive fixation leads. The active fixation leads were associated with reduced risk of pacemaker-related infection.

### Pacemaker-Related Heart Perforation

In the literature, most reported heart perforation events occurred in implantable cardiac defibrillator [18] patients with the correlation between heart perforation and lead characteristics usually being studied in implantable cardiac defibrillator patients [19]. In PM patients, no large cohort study has ever been conducted to investigate the clinical impact of lead characteristics on heart perforation, except for some series of case reports [12, 20–22]. In general, the active fixation type was reported to be a risk factor of PM-related heart perforation [13,21,22]. Furthermore, other than the patient’s characteristics, other reported risk factors for PM-related heart perforation include the location of lead tip [22], temporal pacemaker lead [14], and the operator experience. Although the details of procedures, such as tip location, were not available in this study, some case control studies showed that the location of tip had no statistically significant impact on incidence of heart perforation [13,22]. In this study, we evaluated the relationship between the characteristics of leads and heart perforation after adjusting for all patient characteristics, medication, and operator’s volume, and we found that both the fixation type and insulation materials of leads did not differ in the incidence of either early or late heart perforation.

### Pacemaker-Related Infection

Pacemaker-related infection has attracted more attention and many studies have investigated the risk factors of PM-related infection. In some research, PM-related infection was sub-grouped into early and late infection and risks related to early and late infection were found to be different [23]. Among the well-known risks [7–11, 23–25] all procedure-related factors contributed to the early PM-related infection and some comorbidities were associated with late infection. Unfortunately, the characteristics of leads have been paid scant attention in PM-related infection. In our study, we investigated the relationship between the characteristics of lead and early/late infection and whether or not the infection necessitated lead extraction. We found that the majority of PM-related infection events were early infection, and the PM-related cumulative infection rate was significantly lower in the active fixation group compared to the both fixation group and the passive fixation group. In the subgroup analysis, the active fixation group had a lower early PM-related infection rate and a lower rate of PM-related infection that required lead extraction compared to either the both fixation group or the passive fixation group. Other than active fixation leads, we could not exclude some procedure-related risk factors that might have impacted on PM-related infection. However, we were unable to obtain the details of the procedure in this insurance database. Further studies are thus warranted to examine the biological mechanisms of our observations.

### Limitations

This retrospective analysis bears the inherent limitations of these types of studies. One major limitation was that procedure details could not be obtained, since data were from an insurance system where diseases were classified according to ICD-9 code (Table 1), and payment was just made according to procedure type. Therefore, procedure-related variables, which might affect the early complications, could not be analyzed, thus possibly introducing some bias. In the analysis of the early infection, such issues did in fact exist. Further study should be conducted to confirm these observations that included procedure details in the analysis. Another limitation is that the severity of the complications could not be obtained in this insurance database, and some complications may have been underestimated. Nevertheless, the complications requiring treatment were more important clinically than those that required no therapy.

## Conclusions

In bradyarrhythmic patients, there was no difference in heart perforations between active and passive fixation leads, and active fixation leads were associated with reduced risk of pacemaker-related infection.

## Supporting Information

S1 Table ICD-9-CM code used for diagnosis or treatment. (DOCX)

S2 Table Number of events (time to event, %) by year and lead fixation method. (DOCX)

S3 Table Number of events (time to event, %) by year and lead insulation. (DOCX)
