# Supplementary material for: Impact of Pacemaker Lead Characteristics on Pacemaker Related Infection and Heart Perforation: A Nationwide Population-Based Cohort Study
Source: PLoS One. 2015 Jun 15;10(6):e0128320. doi: 10.1371/journal.pone.0128320 (PMC4468132; doi:10.1371/journal.pone.0128320)
Supplement: S1 Table — (DOCX) [file pone.0128320.s001.docx]

**S1 Table.** I**CD-9-CM Code Used for Diagnosis or Treatment**

| **Variable** | **Source** | **Code** |
| --- | --- | --- |
| AV block | ICD-9 CM code | 4260–4261, 42610–42613, 42654, 4266 |
| Congenital AV block | ICD-9 CM code | 74686 |
| Atrial fibrillation | ICD-9 CM code | 42731 |
| Sick sinus syndrome | ICD-9 CM code | 42781 |
| Diabetes | ICD-9 CM code | 250 |
| Liver cirrhosis | ICD-9 CM code | 5712, 5715, 5716 |
| Obstructive lung disease | ICD-9 CM code | 491–493 |
| Chronic kidney disease | ICD-9 CM code | 585 |
| Heart failure | ICD-9 CM code | 428 |
| Hypertension | ICD-9 CM code | 401–404 |
| Coronary artery disease | ICD-9 CM code | 413,4140 |
| Heart perforation | ICD-9 OP code | 374 |
| PM infection | ICD-9 CM code | 99661, 99662, 99660 |

AV block = atrioventricular block; PM infection = pacemaker infection
