# Supplementary material for: Impact of Pacemaker Lead Characteristics on Pacemaker Related Infection and Heart Perforation: A Nationwide Population-Based Cohort Study
Source: PLoS One. 2015 Jun 15;10(6):e0128320. doi: 10.1371/journal.pone.0128320 (PMC4468132; doi:10.1371/journal.pone.0128320)
Supplement: S2 Table — (DOCX) [file pone.0128320.s002.docx]

**S2 Table. Number of Events (Time to Event,** %**) by Year and Lead Fixation Method**

|  |  | **Infection** –– no. (%^＊^) | | | **Heart Perforation** –– no. (%^＊^) | | |
| --- | --- | --- | --- | --- | --- | --- | --- |
| Year | Patient –– no. | Active fixation | Passive fixation | Both fixation | Active fixation | Passive fixation | Both fixation |
| 1997 | 1,580 | 0 (0.00) | 15 (0.95) | 0 (0.00) | 0 (0.00) | 0 (0.00) | 0 (0.00) |
| 1998 | 1,755 | 0 (0.00) | 14 (0.80) | 0 (0.00) | 0 (0.00) | 0 (0.00) | 0 (0.00) |
| 1999 | 2,094 | 0 (0.00) | 10 (0.48) | 0 (0.00) | 0 (0.00) | 0 (0.00) | 0 (0.00) |
| 2000 | 1,991 | 0 (0.00) | 21 (1.06) | 0 (0.00) | 0 (0.00) | 5 (0.25) | 0 (0.00) |
| 2001 | 2,283 | 0 (0.00) | 15 (0.66) | 0 (0.00) | 0 (0.00) | 2 (0.09) | 0 (0.00) |
| 2002 | 2,377 | 0 (0.00) | 16 (0.69) | 0 (0.00) | 0 (0.00) | 1 (0.04) | 0 (0.00) |
| 2003 | 2,013 | 0 (0.00) | 12 (0.73) | 3 (1.14) | 0 (0.00) | 0 (0.00) | 0 (0.00) |
| 2004 | 2,148 | 0 (0.00) | 6 (0.39) | 1 (0.23) | 0 (0.00) | 1 (0.06) | 0 (0.00) |
| 2005 | 2,145 | 0 (0.00) | 4 (0.25) | 3 (0.71) | 1 (0.68) | 0 (0.00) | 0 (0.00) |
| 2006 | 2,411 | 0 (0.00) | 15 (0.85) | 2 (0.47) | 0 (0.00) | 0 (0.00) | 0 (0.00) |
| 2007 | 2,325 | 2 (0.49) | 10 (0.66) | 6 (1.50) | 0 (0.0) | 0 (0.0) | 0 (0.0) |
| 2008 | 2,925 | 1 (0.18) | 16 (0.85) | 3 (0.61) | 0 (0.0) | 0 (0.0) | 1 (0.2) |

^＊^：the denominator was patient number of different group in different year
