# Supplementary material for: Impact of Pacemaker Lead Characteristics on Pacemaker Related Infection and Heart Perforation: A Nationwide Population-Based Cohort Study
Source: PLoS One. 2015 Jun 15;10(6):e0128320. doi: 10.1371/journal.pone.0128320 (PMC4468132; doi:10.1371/journal.pone.0128320)
Supplement: S3 Table — (DOCX) [file pone.0128320.s003.docx]

**S3 Table. Number of Events (Time to Event,** %**) by Year and Lead Insulation**

|  |  | **Infection** –– no. (%^＊^) | | | **Heart Perforation** –– no. (%^＊^) | | |
| --- | --- | --- | --- | --- | --- | --- | --- |
| Year | Patient –– no. | Silicone | Polyurethane | Both | Silicone | Polyurethane | Both |
| 1997 | 1,580 | 15 (0.96) | 0 (0.00) | 0 (0.0) | 0 (0.00) | 0 (0.00) | 0 (0.00) |
| 1998 | 1,755 | 13 (0.75) | 0 (0.00) | 1 (4.17) | 0 (0.00) | 0 (0.00) | 0 (0.00) |
| 1999 | 2,094 | 10 (0.48) | 0 (0.00) | 0 (0.0) | 0 (0.00) | 0 (0.00) | 0 (0.00) |
| 2000 | 1,991 | 21 (1.06) | 0 (0.00) | 0 (0.0) | 5 (0.25) | 0 (0.00) | 0 (0.00) |
| 2001 | 2,283 | 15 (0.67) | 0 (0.00) | 0 (0.0) | 2 (0.09) | 0 (0.00) | 0 (0.00) |
| 2002 | 2,377 | 8 (0.38) | 4 (2.47) | 4 (2.94) | 1 (0.05) | 0 (0.00) | 0 (0.00) |
| 2003 | 2,013 | 12 (0.84) | 3 (0.62) | 0 (0.0) | 0 (0.00) | 0 (0.00) | 0 (0.00) |
| 2004 | 2,148 | 4 (0.30) | 3 (0.50) | 0 (0.0) | 0 (0.00) | 1 (0.17) | 0 (0.00) |
| 2005 | 2,145 | 5 (0.35) | 2 (0.35) | 0 (0.0) | 0 (0.00) | 1 (0.18) | 0 (0.00) |
| 2006 | 2,411 | 12 (0.70) | 4 (0.74) | 1 (0.67) | 0 (0.00) | 0 (0.00) | 0 (0.00) |
| 2007 | 2,325 | 15 (0.89) | 1 (0.26) | 2 (0.75) | 0 (0.0) | 0 (0.0) | 0 (0.0) |
| 2008 | 2,925 | 14 (0.66) | 4 (1.39) | 2 (0.39) | 1 (0.05) | 0 (0.0) | 0 (0.0) |

^＊^：the denominator was patient number of different group in different year
